# Supplementary material for: Identification of photoperiod-induced specific miRNAs in the adrenal glands of Sunite sheep (Ovis aries)
Source: Front Vet Sci. 2022 Jul 22;9:888207. doi: 10.3389/fvets.2022.888207 (PMC9354845; doi:10.3389/fvets.2022.888207)
Supplement: Supplementary file 1 [file Data_Sheet_1.ZIP › Supplementary file/miRDeep2 file.zip!/miRDeep2 file/Know_Structure/LP42A2/oar-mir-3956.pdf]

miRBase precursor : oar-mir-3956
Total read count : 127
oar-miR-3956-5p read count : 125
oar-miR-3956-3p read count : 2
remaining reads : 0

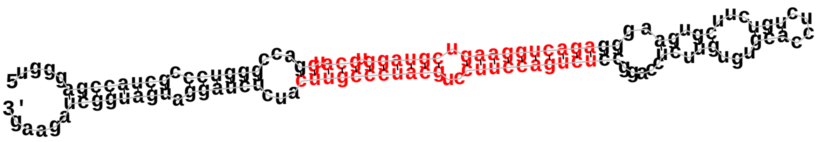

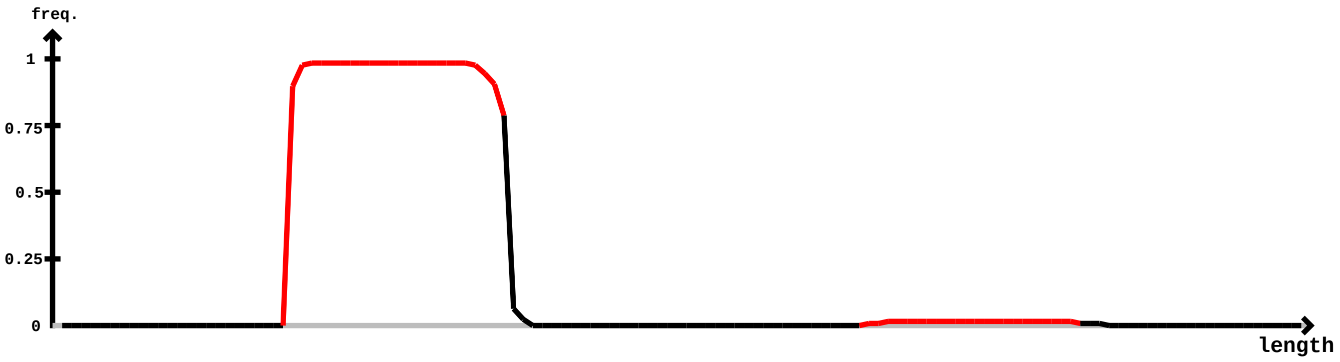


```
                                                                                    oar-miR-3956-3p
                       oar-miR-3956-5p
5'- ugggagccaucgcccugggccagguacguggaugcugaaggucagagggaagugcuucugucuccacguguguucuccaggccucugaccuuccugcaucccguucaucucuaggaugauggcuagaag   -3'    exp
    ....((((((((.((((((...((.(((.((((((.((((((((((((..((.((...(((....)))...)).)).....))))))))))))..)))))))))))...)))))).))))))))....     reads      mm
    .......................guacguggaugcugaaggu.......................................................................................     1          0
    .......................guacguggaugcugaagguc......................................................................................     4          0
    .......................guacguggaugcugaagguca.....................................................................................     5          0
    .......................guacguggaugcugaaggucag....................................................................................     13         0
    .......................guacguggaugcugaaggucaga...................................................................................     84         0
    .......................guacguggaugcugaaggucagag..................................................................................     4          0
    .......................guacguggaugcugaaggucagagg.................................................................................     3          0
    ........................uacguggaugcugaaggucag....................................................................................     2          0
    ........................uacguggaugcugaaggucaga...................................................................................     8          0
    .........................acguggaugcugaaggucagag..................................................................................     1          0
    ...................................................................................ucugaccuuccugcaucccguu........................     1          0
    .....................................................................................ugaccuuccugcaucccguucau.....................     1          0
```
